# The association between hordein polypeptide banding and agronomic traits in partitioning genetic diversity in six-rowed Ethiopian barley lines (*Hordeum vulgare* L.)

**DOI:** 10.1186/s12870-023-04117-x

**Published:** 2023-02-20

**Authors:** Basazen Fantahun, Tesfaye Woldesemayate, Eleni Shiferaw

**Affiliations:** Ethiopian Biodiversity Institute, P.O.Box. 30726, Addis Ababa, Ethiopia

**Keywords:** Barley, Genetic diversity, Hordein, SDS-PAGE, Multiple regression

## Abstract

**Background:**

Evaluation of the extent of genetic variation within and between the populations of crop genetic resources are of paramount importance in any breeding program. An experiment aimed at assessing the extent of variation among barley lines and the degree of association between hordein polypeptide and agronomic traits was hence executed.

**Methods:**

Field experiment was conducted in six environments between 2017–2019 involving 19 barley lines. Hordein bands were separated using vertical Sodium Dodecyl Sulphate Poly- acrylamide Gel Electrophoresis (SDS-PAGE).

**Results:**

The analysis of variance revealed significant variation among lines and wider range units were observed for the agronomic traits. The line (Acc# 16,811–6) was superior, producing the highest grain yield (2.97 ton ha^−1^) across environments, 3.6 ton ha^−1^ at Holleta, and 1.93 ton ha^−1^ at Chefedonsa. At Arsi Negelle a different line Acc# 17146–9 was the highest yielding (3.15ton ha^−1^). SDS-PAGE-based analysis of barley lines separated 12 hordein bands between C (four bands) and B (eight bands) subunits. Interestingly bands 52, 46a, and 46b were uniquely conserved in the four naked barley lines (Acc#16809–14,16956–11, 17240–3, 17244–19). A considerably high proportion of genetic diversity within the populations than among the populations could be a repercussion of high gene flow which substantiates the longstanding and dominant informal seed exchange system among the farmers. The significant positive association between grain yield and band 50 evocates the expression of this allele may code for higher grain yield. The negative association between days to maturity and band 52 perhaps stipulates earliness in barely lines upon the manifestation of the band. Band 52 and 60 appeared to be associated with more than one agronomic trait (days to maturity and thousand kernel weight; grain filling period and grain yield respectively) and could be the result of pleiotropic characteristics of the genes residing in these banding regions.

**Conclusion:**

The barley lines exhibited substantial variation for hordein protein and agronomic traits. However, imparted the need for the implementation of decentralized breeding as a consequence of genotype-by-environment interaction. Significant hordein polypeptide and agronomic traits association advocated the utilization of hordein as a protein marker and perhaps consider them in the parental line selection.

**Supplementary Information:**

The online version contains supplementary material available at 10.1186/s12870-023-04117-x.

## Background

To put the most effective and efficient crop improvement program in place, access to crop genetic resources is quite fundamental. The characterization of these crop genetic resources for different agro-morphologic traits and the evaluation of the extent of genetic variation within and between the populations are most pivotal in any breeding program [1]. More specifically in the identification of parental materials for crossing programs to attain better gene combinations and in the recommendation of cultivars with desirable traits, the variation in the already available genetic resources is essential. In barley, genetic diversity as in most field crops could be analyzed based on agro-morphologic traits [2–5]; based on biochemical markers [6, 7], and/or based on molecular markers [8, 9]. Diversity analysis based on agro-morphological markers perse may be hampered by limited specificity and resolution of the markers and genotype by environment interaction [10]. To this end, the application of biochemical markers is believed to complement such limitations especially hordein proteins in barley serve as protein markers as they are tolerant to mutations [11].

Proteins in barley grain can be separated into albumin, globulin, prolamin (hordein), and gluten fractions [12]. The hordeins are alcohol-extractable protein fractions of barley that constitute 35–50% of the total protein content [13]. Components of the hordein fraction are classified into three groups of polypeptides B, C, and D in order of decreasing mobility [14]. The B and C fractions account for 70–80% and 10–12%, respectively [15] whereas the D fractions constitute only 5% [16]. These fractions of hordeins differ in molecular weight and amino acid composition [17] and are categorized as sulfur-rich (B-hordeins), sulfur-poor (C-hordeins), and high molecular weight (HMW, D-hordeins) [17]. The B and C groups of polypeptides are controlled by complex loci, designated as *Hor-2*, and *Hor-1* respectively [18]. *Hor-1* and *Hor-2* are located on the short arm of chromosome 5, 1H [18, 19]. The D hordein is identified for its high glycine, proline and glutamine content. This group of hordein is coded by the *Hor 3* locus located on the long arm of chromosome 5 [16]. Hordein is known for great inter-genotypic variation and has been used as a marker in cultivar identification, genetic diversity studies and in determining phylogenic origins [7, 18, 20, 21]. SDS-PAGE technique has been applied for separating proteins, examining the biochemistry and genetics of hordein to describe the genetic structure of crop germplasm [22]. This method has been reported as an alternative screening test to differentiate cultivars [23]. Relation between agronomic traits and hordein protein was reported in [24, 25] based on correlation analysis among the characters and based on regression analysis [26]; between hordein and malt quality traits [23, 27, 28]; between hordein and RAPD markers [29]. Little effort has been made to identify the association between hordein protein and agronomic traits in Ethiopian barley. In the present study agro-morphologic traits and SDS-PAGE analysis of hordein polypeptide were used to analyze the genetic diversity among 19 barley lines. The study aimed to assess the diversity among the barley lines for agro-morphologic and hordein protein; determine the association between agro-morphologic and hordein polypeptide bands.

## Results

### Agronomic characters variance component analysis

The REML variance component analysis was employed in an attempt made to dissect the phenotypic diversity among the barley lines. The same analysis showed that there was a very highly significant variation (*p* < *0.001*) among the barley lines for all characters under scrutiny pooled over the six environments (Table 1). The only exception to this was the number of effective tillers that showed significant variation at *p* < *0.05*. Similarly, environmental and genotype-by-environment interaction effects had shown that there was highly significant variation at *p* < *0.001* for all the traits. The barley lines in the study exhibited considerable variation as expressed by maximum and minimum mean performance values, range unit, and grand mean estimated based on the pooled data for the traits under study (Table S1). Grain yield in this case showed the widest range spreading between 1.87–2.97 ton ha^−1^ with 1.11 ton ha^−1^ range values and mean performance of 2.51 ± 0.03. PHT extended between (92.26–104.40 cm) with 12.14 cm range units, DH (69.74–81.16 days; range unit of 11.42 days), DM (107.65–117.44 days; range unit of 9.79 days) and the other traits revealed relatively lower range unit.

**Table 1:** Variance component analysis of nine agronomic traits based on 19 barley lines combined over six environments

Traits	Source of variation				Grand mean	LSD	CV (%)
	Environment variance	Genotype variance	Genotype x Env. variance	Error variance			
DH	118.96^***^	12.25^***^	13.84^***^	3.02	75.62	3.19	2.30
DM	159.85^***^	11.37^***^	12.94^***^	3.23	112.57	3.09	1.60
GFP	6.75^***^	1.93^***^	9.51^***^	3.82	36.94	1.96	5.29
PH (cm)	32.69^***^	10.99^***^	18.99^***^	6.88	98.05	3.48	2.68
NET	0.42^***^	0.01^*^	0.10^***^	0.11	2.08	0.16	16.12
SPL (cm)	0.36^***^	0.26^***^	0.24^***^	0.16	6.12	0.44	6.44
NK	27.79^***^	8.98^***^	17.55^***^	6.62	46.76	3.27	5.50
TKW (gm)	5.02^***^	9.07^***^	10.56^***^	6.37	38.87	2.77	6.49
GY (t ha^−1^)	0.30^***^	0.14^***^	0.11^***^	0.03	2.51	0.30	6.45

*DH* Days to heading, *DM* Days to maturity and *GFP* Grain filling period, *PH* Plant height, *NET* Number of effective tillers, *NK* Number of kernel per spike, *SPL* Spike length, *TKW* Thousand kernel weight, *GY* Grain yield, *LSD* Least significant difference, *CV* Coefficient of variation

^***^ = significant at *(P<0.001)* and ^*^=significant at *(P<0.05)*

**Fig. 1:**
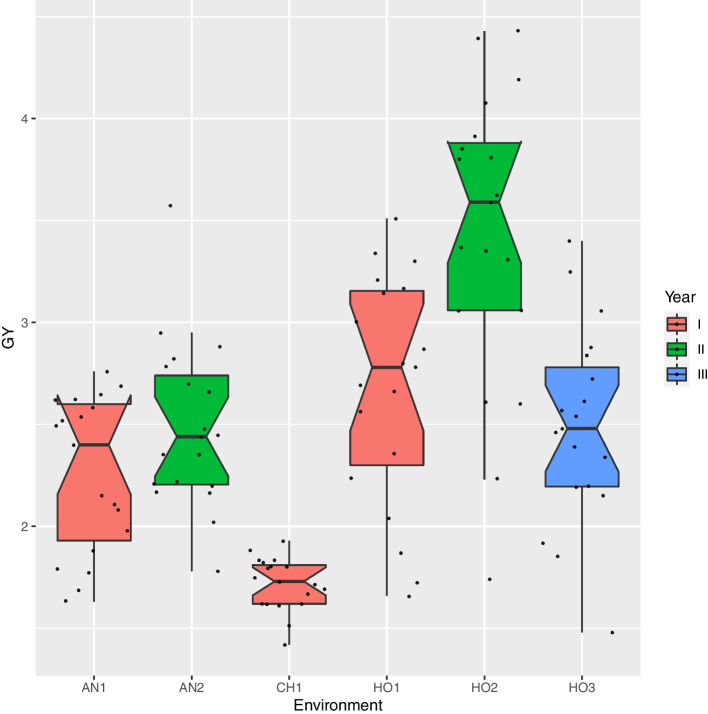
Grain yield with the respect to the six test environments. The x-axis represents, the environments AN1 = Arsi Negelle year I and AN2 = Arsi Negelle year II; CH1 = Chefedonsa year I; HO1 = Holleta year I, and HO2 = Holleta year II. The y-axis represents the estimated grain yield in ton ha^−1^

The distributions of mean performance of the genotypes (BLUP) for GY in each of the environments are described in boxplots (Fig. 1). Amidst all the environments, the highest mean and spread of the BLUPs for GY was observed at Holleta year II and in contrast, the lowest BLUP values were detected at Chefedonsa year I.

The genotype (Acc# 16811–6) was found to be a stunning line with respect to grain yield producing the highest yield (2.97ton ha^−1^) across environments, 3.6 ton ha^−1^ at Holleta and 1.93ton ha^−1^ at Chefedonsa (Table S2-1). At Arsi Negelle Acc# 17146–9 was the highest yielding line (3.15 ton ha^−1^) and when combined over the environments the same line with a mean grain yield of 2.84 ton ha^−1^ had no significant difference compared to the best line Acc# 16811–6. The mean performance of pheno-agronomic traits other than GY combined across the environments is also presented in Table S2-2.

### Hordein polypeptide banding pattern-based diversity analysis

**Fig. 2:**
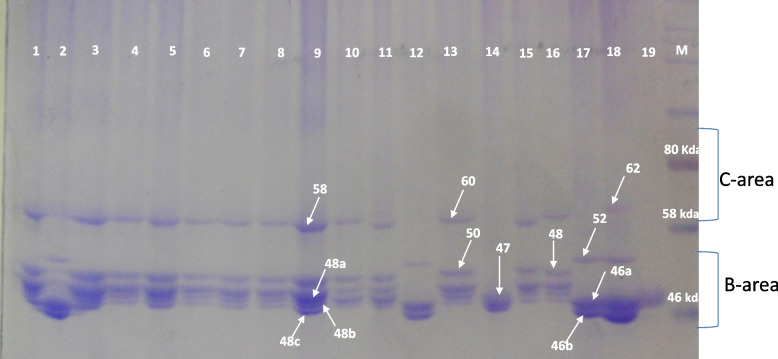
Hordein polypeptides migration patterns in 19 six-rowed barley lines based on SDS gel electrophoresis from a single seed of barley lines, M = molecular weight makers with the molecular weight (kDa)

SDS-PAGE-based analysis of 18 barley lines derived from farmers’ varieties and an improved variety identified 12 hordein bands between C and B subunits which are controlled by *Hor 1* and *Hor 2* locus respectively, located on the short arm of chromosome 5. These bands were spanning in the molecular weight regions between 46 to 62 kDa (Fig. 2) and they were further separated into their respective subunits in which case four of the bands were residing on the C-subunit and the remaining eight were residing in the B-subunit.

The identified hordein bands showed nine distinct polypeptide banding patterns (Table 2). Among the lines sifted, seven had unique migration patterns and banding pattern 1 was found to be the most common, occurring in 47.36% of the lines. The majority of the barley lines (12/19) had the highest number of bands (five) and the line Acc# 17148–16 and the cultivar HAR-1307 had the least number of bands (one). The number of bands in each banding pattern varied between one (patterns 5 and 9) and five (patterns 1,4,6 and 7).

**Table 2:** Hordein banding of 19 six-rowed Ethiopian barley lines with respective geographic origin, caryopsis type, bands and banding pattern

TRT	Genotypes	Geography of origin	Caryopsis type	Bands	Banding pattern
1	16734–6	Guraghe	Hulled	58,48,48a,48b,48c,	1
2	16809–14	Hadiya	Hulless	52,46a,46b	2
3	16810–13	Hadiya	Hulled	58,48,48a,48b,48c,	1
4	16811–6	Hadiya	Hulled	58,48,48a,48b,48c,	1
5	16812–4	Hadiya	Hulled	58,48,48a,48b,48c,	1
6	16814–7	Hadiya	Hulled	58,48,48a,48b,48c,	1
7	16820–16	Guraghe	Hulled	58,48,48a,48b,48c,	1
8	16822–12	Guraghe	Hulled	58,48,48a,48b,48c,	1
9	16824–15	Guraghe	Hulled	58,48,48a,48b,48c,	1
10	16863–2	Arsi	Hulled	60,48,48a,48b,48c	3
11	16910–19	Arsi	Hulled	58,48,48a, 48b, 48c	1
12	16956–11	Arsi	Hulless	52,46a,46b	2
13	17146–9	Arsi	Hulled	60,50,48a,48b,48c	4
14	17148–16	Arsi	Hulled	47	5
15	17204–5	North Gonder	Hulled	58,50,48a, 48b,48c	6
16	17206–11	North Gonder	Hulled	60,48,48a,48b,48c	7
17	17240–3	Awi	Hulless	52,46a, 46b	2
18	17244–19	Awi	Hulless	62,52,46a,46b	8
19	HAR-1307	Improved	Hulled	48c	9

In the current experiment we report the frequency of appearance of particular band across the 19 barley lines (the number of times a given band appeared) and the frequency of a banding pattern which shows how often the same group of bands occurred across the lines. Out of the four bands at the *Hor-1* locus three (bands 60, 58 and 52) were common with frequencies > 0.100 and all the bands except bands 50 and 47 at the *Hor-2* locus occurred at higher frequencies (Table 3). The band 48c at the B subunit occurred at a dominantly high frequency (0.737). The hordein-based banding pattern didn’t follow the geographic origin of the lines in which case either lines collected from different zones (administrative structure below a region) showed similar hordein banding patterns or lines from the same zone revealed varied banding patterns. The exception to this was the lines from the Guraghe zone where all the lines had the same banding pattern. As opposed to this, all five lines from Arsi had different banding patterns. Interestingly bands 52, 46a, and 46b were unique to the four naked barley lines (16809–14,16,956–11, 17240–3, 17244–19) included in the experiment.

**Table 3:** Frequency of hordein polypeptide bands and banding pattern based on 19 six-rowed barley lines

Band	**Band** Frequency	Banding pattern	**Banding pattern** Frequency
Band 62	0.053	1	0.474
Band 60	0.158	2	0.158
Band 58	0.526	3	0.053
Band 52	0.211	4	0.053
Band 50	0.105	5	0.053
Band 48	0.632	6	0.053
Band 48a	0.684	7	0.053
Band 48b	0.684	8	0.053
Band 48c	0.737	9	0.053
Band 47	0.053		
Band 46a	0.158		
Band 46b	0.158		

**Fig. 3:**
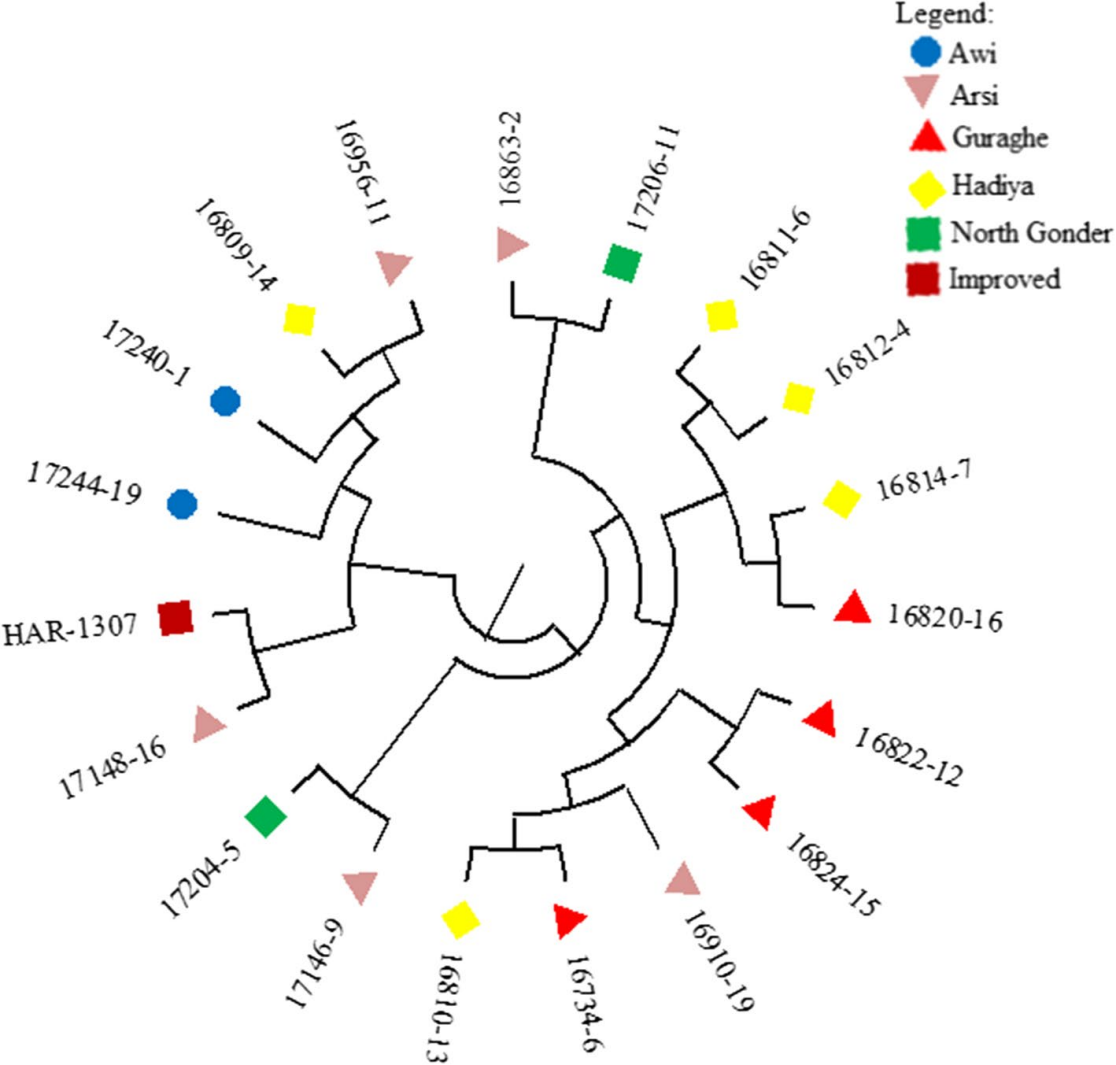
Phylogenetic relationship among 19 barley lines based on unweighted pair group method where the geographic origin of the lines was as per the legend

The estimation of the coefficient of Nei’s genetic similarity between the populations of the barley lines revealed the lowest similarity index was observed between the barley population from Guraghe and Awi (0.468) and the highest was between Guraghe and Hadiya (0.977) with an average of 0.757 across all the population (Table S3, Fig. 3).

Within the population the estimates of parameters of genetic diversity, mean number of alleles per locus (*N*_a,_), effective number of alleles (*N*_e_), Shannon diversity index (*H*), percentage of polymorphic loci (*p%*), mean genetic diversity (*h*_e_) span between 0.250–1.333 (*N*_a,_), 1.000–1.620 (*N*_e,_), 0.000–0.53 (*H*), 0.00–91.67 (*p%*), 0.042–0.360 (*h*_e_) (Table 4).

**Table 4:** Within population genetic diversity estimates based on hordein protein for 19 six-rowed barley lines

Population ID	Mean number of alleles per locus (Na)	Effective No of alleles (Ne)	Shannon’s diversity Index (H)	Percentage of polymorphic loci (p%)	Mean Genetic diversity (h)
Awi	0.250	1.083	0.058	8.33	0.042
Arsi	1.833	1.620	0.531	91.67	0.360
Guraghe	0.417	1.000	0.000	0.00	0.000
Hadiya	1.333	1.314	0.334	66.67	0.213
North Gonder	0.917	1.333	0.231	33.33	0.167
Mean				40.00	
SE				17.36	

Furthermore, considerably high gene flow (1.588) was also observed among the barley lines. The AMOVA results highlighted, a greater proportion of genetic variance was observed within the populations, which accounted for 76%, whereas among populations the recorded variance was 24% (Table 5).

**Table 5:** Analysis of molecular variance (AMOVA) based on hordein protein for 19 six-rowed barley lines

Source of variation	Degree of Freedom	Mean Square	Estimated Variance	Percentage of variance (%)	Probability level
Among Pops	4	3.17	0.47	24%	0.001
Within Pops	13	1.51	1.51	76%	0.001
Total	17		1.99	100%	

### Association between agronomic traits and hordein polypeptide bands

In an attempt made to unpack the degree of association among the agronomic traits and biochemical makers, multiple regression was run. For this analysis, nine agronomic traits (each as a dependent variable) and 12 hordein bands (independent variables) were included. Before running the regression analysis, however, Pearson correlation was carried out to diagnose whether there was a collinear relation among the independent variables (Table S4). By doing so it was found that six of the independent variables had collinear characteristics. Therefore, bands 62, 60, 58, 50 and band 48c were retained in the analysis and each of the agronomic traits was regressed over these variables (hordein bands). The significant regression analysis result unveiled that there were statistically supported associations between the hordein polypeptide bands and agronomic traits at *p* < 0.05 for days to maturity, grain filling period, grain yield and thousand kernel weight (Table 6). It was also shown that the hordein polypeptide bands in the multivariate regression model had explained up to 73.31% (R^2^) of the variation in the dependent variable, thousand kernel weight in this case. The degree of association among the characters that exhibited significant association with the hordein bands spanned between 61.1% (GFP) and 73.1% (TKW).

**Table 6:** Regression analysis of variance among eight agronomic traits and six hordein polypeptide bands at *p* < *0.05*

	DF		MS		F	*p*-value	R^2^	SE
	Regression	Residual	Regression	Residual	Regression	Regression		
DH	6	12	18.136	6.226	2.913	0.054	0.593	2.495
DM	6	12	18.139	5.072	3.576	0.029^*^	0.641	2.252
GFP	6	12	1.833	0.584	3.141	0.043^*^	0.611	0.764
PHT	6	12	8.164	8.385	0.974	0.483	0.327	2.896
TIL	6	12	0.004	0.002	1.968	0.150	0.496	0.044
SPL	6	12	0.303	0.169	1.793	0.183	0.473	0.411
NK	6	12	10.254	4.719	2.172	0.119	0.521	2.172
TKW	6	12	16.142	2.970	5.435	0.006^**^	0.731	1.723
GY	6	12	0.263	0.049	5.394	0.006^**^	0.730	0.221

^*^ = significant at *p* < *0.05*, ^**^ = significant at *p* < *0.01*

In the dissection of the contribution of each of the hordein bands (independent variables) with respect to each of the agronomic characters, we used the same analysis (regression). The analysis uncovered that among the six predictor variables (hordein bands) four were significantly associated with agronomic traits (Table 7). Distinctively, band 52 was found to be more important in explaining the variation in DM and TKW, band 60 in explaining the variation in GFP and GY, band 58 in explaining GFP and band 50 in explaining GY as they were statistically significant at *p* < 0.05.

**Table 7:** Hordein polypeptide bands showed significant explanatory value at *p* < 0.05 with agronomic traits that were significant in the general regression analysis

	Band	Coefficient	Standard error	*p*-value
DM	Band 52	-8.413	2.600	0.007
GFP	Band 60	-2.668	0.905	0.012
	Band 58	-2.860	0.804	0.004
GY	Band 60	-0.613	0.262	0.037
	Band 50	0.407	0.177	0.039
TKW	Band 52	-7.583	1.990	0.002

Stepwise regression analysis was applied to further identify those hordein bands that should remain in the model to best explain the association. The analysis was done in such a way that it combined forward selection and backward elimination. The level of significance used in forward selection (penter) was 0.05 and for backward elimination (prem) was 0.1. With this procedure, GY was best explained by three bands 50, 52 and 60. For GFP among the two bands that showed a significant association, only band 58 remained in the model, band 60 was eliminated from the model after stepwise regression. Band 52 remained in the model to best explain DM and TKW (Table 8).

**Table 8:** Hordein bands exhibiting strong association with agronomic characters after stepwise regression analysis

Trait	Variable	Added/Removed	Adj.R-square	C(p)	AIC	RMSE
DM	Band 52	Addition	0.488	1.164	87.698	2.100
GFP	Band 58	Addition	0.163	9.390	54.438	0.915
GY	Band 52	Addition	0.360	11.803	9.047	0.277
	Band 60	Addition	0.552	4.66	3.119	0.232
	Band 50	Addition	0.667	1.309	-1.739	0.200
TKW	Band 52	Addition	0.603	2.696	79.252	1.758

*C(p)* Mallows Cp, *AIC* Akaike Information Criterion, *RMSE* Residual mean square error

## Discussion

The highly significant variation and wider range units among the barley lines as revealed by the analysis of variance were indicative of the existence of the enormous variability among the analyzed barley lines. This perhaps emanates from diverse agro-ecological and cultural states that make Ethiopia to be one of the world’s crop diversity hotspots, barley included [30]. This result accords with the findings of [2, 3, 31–33] who reported significant variation for agronomic traits from 120 barley landraces collected from ‘*Bale*’, 22 landraces from Southern Ethiopia, six improved malt barley genotypes, ten landraces and three cultivars from Jordan, and eight malting barley released varieties from North Ethiopia respectively. Significant variation among the test environment revealed the existence of statistically supported variation among the environments and this was in harmony with the finding of [31]. The REML analysis has also disclosed that all the traits with no exception had highly significant genotypes by environment interaction. This suggested that barley lines had differential responses across environments [3, 5, 34, 35] and this can be considered as one of the prominent contests researchers are facing as it reduces the selection efficiency thereby creating some degree of uncertainty [36].

The experiment was conducted in three locations and six environments in which case Holleta and Chefedonsa were located in the central highland and in contrast Arsi Negelle was located in the rift valley belt which is specifically characterized by terminal moisture stress. Nevertheless, the least performance of the barley lines was observed at Chefedonsa dictating less suitability of the site for barley production. This may conceivably be related to the edaphic factors which classify the soil in this location to be black vertisol imposing water logging property [37]. The barley line Acc# 17146–9 was a significantly high yielder at Arsi Negelle than any of the barley lines including the improved HB-1307. However, this line was not among the best five high-yielding lines at Holleta. Conversely, the best-performing line 16811–6 at Holleta was not within the best five at Arsi Negelle. This result was supportive of the aforementioned finding that indicated significant genotype by environment interaction effect. Expectedly, the best-performing line at Holleta was also found to perform best at Chefedonsa though with the lowest BLUP mean compared to any of the locations.

Genetic variation in a panel of germplasm has an essential role in the identification of varieties. Variation in the electrophoretic banding pattern of proteins is linked with the genotype’s genetic constitution and thus it can be exploited to comprehend the genetic makeup [38]. In the current study, we analyzed genetic variation in six-rowed barley lines using hordein markers representing two loci (*Hor 2 and Hor 1*) in the barley genome in which case 12 distinct bands were separated at these loci out of which four bands were separated at *Hor 1*. According to [39] the Ethiopian region was characterized by a high diversity of *Hor 1* alleles which in turn contributed to a high level of polymorphism in hordein. The detection of nine banding patterns out of which seven were unique indicated that there was an important set of genetic polymorphisms among the barley lines included in the study. The existence of seven unique migration patterns in the electrophoresis further signified the effectiveness of SDS-PAGE electrophoresis as a screening test in discriminating cultivars. This is possibly due to hordein migration pattern is varietal characteristics and being less influenced by the environment [23, 40], to the extent that hordeins are tolerant to mutations [11]. Therefore, hordeins may serve as a genetic marker and are essential in evolutionary studies. Different authors reported a variable number of electrophoretic hordein polypeptide bands and banding patterns. In line with this [29] detected 69 bands from 34 cultivars constituting 15 migration patterns controlled by the B and C subunit. Twenty six hordein polypeptide bands were separated from nine malting and four feeding barley genotypes at B and C fractions [23], the major subunits of hordein that comprises 95% of the total hordein fraction [41]. A total of 37 bands were identified between the slow-moving *Hor 1* and the fast-migrating *Hor 2* giving rise to 53 distinct banding patterns from 53 Lebanese landraces [42]. This variation in the number of hordein polypeptide bands and banding pattern could probably be due to the degree of polymorphism among the varieties included, and the number of varieties. In this experiment, we found three unique bands conserved in the four naked barley lines (one at *Hor 1* and two at *Hor 2*) even though naked barley lines had different geographic origins and these bands were not detected in any of the hulled barley lines. This may be an indicator of the utilization of these bands as a genetic marker for naked caryopsis characteristics in barley and of the effectiveness of SDS-PAGE in differentiating the naked versus hulled barley varieties. However previous findings suggested the gene conferring naked caryopsis in barley, the *nud* gene is located on the long arm of chromosome 7H [43, 44] as opposed to the *Hor 1* and *Hor 2* locus where these unique bands were separated are located on the short arm of chromosome 5 (1H). This may call for further investigation with a larger panel of naked and hulled barley genotypes to confirm this region of the barley genome whether it has any relation with naked caryopsis. In study made to differentiate between spring and winter cultivars [29] reported it was not possible using SDS-PAGE.

The estimation of genetic parameters showed the variation within the four populations was quite different such that the percentage of polymorphic loci ranged from 0.000 to 91.67, and the Shannon diversity index ranged between 0.000 to 0.531 for Guraghe and Arsi populations respectively. This suggested that the lines included in the sampled population from Guraghe showed no variation where all the lines share similar hordein bands. Conversely, all the lines from Arsi responded quite differently with regard to the hordein test conserving no similar hordein bands among each other with few exceptions. In their study on 90 different accessions that comprised four populations collected from Jordan [45] reported percentage polymorphic loci spanning between 79.31–86.21 based on hordein protein. The coefficient of genetic identity among landrace populations was substantially high which ascertains the populations to which the barley lines were belonging were genetically similar. The little genetic differentiation could be the a result of a high level of gene flow detected in these sampled populations [45]. The proportion of gene diversity within the populations was exceedingly greater than among the populations similar to prior reports [8, 9]. The longstanding informal seed exchange system among the local farmers dominating the seed distribution system in the country could be the rationale behind the comparatively high gene flow. Based on the aforementioned facts forthcoming collection missions aimed at addressing untapped geographic origins, characterization and evaluation activities should give more emphasis to accessions within the populations instead of across the populations. Hordeins show high inter-genotypic variation, they are informative markers and the electrophoretic analysis is comparatively low-cost, and easily accessible. This was why we preferred to use these biochemical markers, molecular tools perhaps confer better resolution otherwise.The association analysis based on multiple regression between the agronomic traits and hordein protein showed significant association among the dependent variables (agronomic traits) and the predictor variables (hordein bands). Besides there observed a considerably high magnitude of association as explained by (R^2^). This justifies that some of the observed variations in the agronomic traits could one way or the other be related to the expression of the hordein bands. Such association between hordein polymorphism and variation of agro-morphological traits may unarguably lay a fertile ground for breeders to broaden the germplasm source and support parental choices to be used in their breeding programs [7, 25]. The significant association between the agro-morphologic traits and hordein polymorphism accords with the findings of [25–27] and it was in disagreement with [7].

The detected association between the agronomic traits and hordein polypeptide bands was perhaps the repercussion of linkage among genes conferring a particular agronomic characteristic and a specific hordein banding [26]. It could probably be due to intensive selection pressure towards a certain desirable trait governed by genes in the closer distance with hordein locus. The case in point is the powdery mildew loci located between *Hor 1* and *Hor 2*, hence when selection occurs towards the direction of the powdery mildew resistance allele the chance of indirect selection for some hordein alleles will be very high [29, 46]. Amidst the three bands that best explained GY bands 60 and 52 were negatively associated suggesting the occurrence of these bands can be considered as an indicator for lower grain yield. Band 52 remained in the model after step-wise regression to best explain DM and TKW and this band had also a negative association with these two agronomic traits. This finding unwrapped that the expression of this hordein allele induces earliness to the barley varieties. Earliness is one of the desirable traits that breeders typically look for especially in rain-fed agriculture characterized either by short rainy season or terminal moisture stress, particularly when it combines lower yield penalty of being early. Furthermore, in areas with the bimodal type of rainfall the early varieties are most preferred as these varieties will provide ample land preparation and sowing time for the forthcoming growing season which may be shorter. Band 60 having a significant negative association with GFP and GY further strengthen the fact that conferring shorter grain filling period may induce yield penalty. A significant association between phenologic traits and hordein was reported by [25, 27]. The existence of a given hordein band being associated with more than one agronomic trait and hence affecting these characters could be the outcome of the pleiotropic characteristics of some genes closely linked to the hordein bands [26]. However, in order to confirm these associations and consider it as a genetic marker such results should be augmented with QTL mapping [25].

## Conclusion

The observed variation for agronomic traits in the current study is pivotal to marking parental lines out for hybridization and introgression of desirable traits into the available genetic base in the national breeding programs. Differential response of the barley lines over the test locations rule in the imperativeness of decentralizing the breeding programs and variety release scheme of the country to best exploit the potential of the varieties. The banding pattern based on SDS-PAGE showed hulled and naked barley lines could be differentiated using hordein data. However, there is a need to undertake further study to confirm this finding with a diverse set of barley lines where naked and hulled barley types are included. The presence of unique bands conserved only in the naked caryopsis augments the application of hordein bands as a genetic marker. Markedly high gene flow among the population and a higher proportion of genetic variance within the population than among the populations was additional evidence that supports future barley improvement programs should give due emphasis to variation within the population. The regression analysis reaffirmed that the hordein bands are somehow associated with some of the agronomic traits. This dictated selection to the direction of these bands may either positively or negatively affect the agronomic performance and hence positive associations are considered an effective indicator for parental choice in crossing programs.

## Materials and methods

### Plant material

**Fig. 4:**
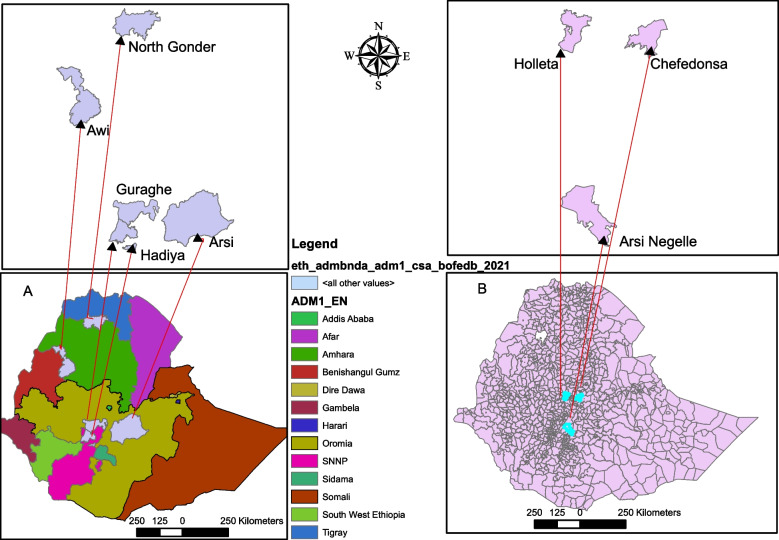
A map of Ethiopia showing the administrative regions and zones where the barley farmers’ varieties included in this study were collected (**A**) and the sites (districts) where the field experiments were carried out (**B**)

In this experiment, 18 barley lines were purified in an ear-to-row fashion from 18 accessions obtained from the Ethiopian Biodiversity Institute (http://www.ebi.gov.et) and an improved variety HB-1307 received from Holleta Agricultural Research Center (http//www.eiar.gov.et) were included. These barley lines represent five different administrative zones among the major barley-growing regions of the country with altitudes ranging between 2500–3200 m.a.s.l. (Table S5) (Fig. 4). These genetic materials are six-rowed spike type of barley lines comprising 15 hulled and four hulless barley.

### Common garden experiment

The experiment was laid according to Randomized Complete Block Design with three replications in six rows of 2.5 m length, 0.2 m row spacing where each plot of 3 m^2^ was sown with 26 g of seeds. The experiment was conducted at three locations during the main cropping season, Holleta (for three years), Arsi Negelle (for two years) and Chefedonsa (for one year) hence the experiment was conducted in six environments between 2017 and 2019. Holleta and Chefedonsa are located in the central highland and Arsi Negelle is located in the African Rift Valley belt. The plots were fertilized with DAP and Urea fertilizers as per the recommended rate of applications for the three sites and the seeds were planted by hand drilling. Planting was carried out in early July at Holleta, in mid-July at Arsi Negelle and in late July at Chefedonsa. Relevant crop management practices were carried out following the standard agronomic recommendations.

### Data collection

Data collection was based on the descriptor list developed by Bioversity International [47]. Three phenological and six agronomic traits were collected in the current experiment. The phenological characters were days to heading (DH), and days to maturity (DM); the agronomic traits were plant height (PHT), number of effective tillers (NET), spike length (SPL), number of kernel per spike (NK), grain yield (GY) and thousand kernel weight. The phenological traits, grain yield and thousand kernel weight (TKW) were collected on a plot basis. Data on plant height, number of effective tillers, spike length and number of kernels per spike on the other hand were collected from five randomly sampled plants per plot. The data on grain yield and thousand kernel weight was adjusted to 12.5% moisture content.

Hordein separation was conducted using vertical (SDS-PAGE) from the grain harvest of 2019. Seed storage proteins are known to be highly stable and genetically fixed hence seeds only from Holleta site third year were used for hordein analysis. A barley flour sample was obtained by grinding individual seeds. Extraction was done by mixing 0.5 gm of barley flour and 200 µl extraction buffer (55% propanol, 2% β-mercaptoethanol, 1% acetic acid) and keeping it for 1 h at 60^0^c followed by centrifugation for 10 min at 14krpm. The supernatant was then transferred to a clean tube and an equal amount of gel loading buffer (50 mM Tris–HCl (pH6.8), 2% SDS, 0.1% bromophenol blue, 10% glycerol) was added to the supernatant. The separation was done using 10% acrylamide gel (acrylamide (10%), Tris–HCl (pH 8.8), 10% SDS, TEMED and 10% APS) and 5% stacking gel (5% acrylamide, Tris–HCl (pH 6.8), SDS, APS and TEMED. The extracted hordein from each sample was loaded in the gel and a protein ladder (NEB, UK) was included to estimate the molecular weight of hordeins separated by electrophoresis. The gel was run at 100 V-constant power until the indicator dye reaches the bottom of the separating gel. The gel was stained overnight using a staining solution containing 0.25 gm Coomassie blue diluted in 100 ml ethanol, 100 ml distilled water and 25 ml acetic acid. Destaining was done for 12 h using a mixture of ethanol and distilled water (1:1) and the gel was photographed by a digital imaging system. The hordein banding pattern was then determined by scoring the presence and absence of all examined bands. The naming of the bands was done in relation to their position with the respect to the protein ladder.

### Data analysis

The analysis of variance (ANOVA) was based on the restricted maximum likelihood (REML) algorithm and REML was used to produce the best linear unbiased prediction (BLUP) mean. The BLUP means combined over the six environments were calculated using the META- R statistical software version 6.04 [48] and these means were used in subsequent regression analysis. R version 4.1.2 was applied to produce a boxplot (to compare the grain yield over environments and locations) using the *ggplot-2* function [49]. Pooled analysis was run after conducting Hartley’s F-max-based error variance homogeneity test [50]. The REML analysis was carried out using the following model

$${\mathrm{Y}}_{\mathrm{ijk}} =\upmu + {\mathrm{Env}}_{\mathrm{i}} + {\mathrm{Rep}}_{\mathrm{j}}(\mathrm{Env}_{\mathrm{i}}) + {\mathrm{Gen}}_{\mathrm{l}} + {\mathrm{Env}}_{\mathrm{i}}\mathrm{ x }{\mathrm{Gen}}_{\mathrm{l}} + {\varepsilon}_{\mathrm{ijkl}}$$

where Y_ijk_ is the trait of interest, µ is the general mean, Env_i_ is the effect of the _i_th environment, Rep_j_(Env_i_) is the effect of the _j_th replicate within the _i_th environment, Block_k_(Env_i_Rep_j_) is the effect of the _k_th incomplete block within the _i_th environment and _j_th replicate, Gen_l_ is the effect of the _l_th genotype, Env_i_ x Gen_l_ is genotype by environment interaction and Ꜫ_ijkl_ is the error associated with the _i_th environment, _j_th replication, _k_th incomplete block and the _l_th genotype.

Bands of hordein protein scored as present (1) or absent (0) were used for statistical analysis. The software GenAlEx version 6.5 [51] was used to calculate the genetic diversity of each population by estimating the genetic diversity parameters, the mean number of alleles per locus (Na), the effective number of alleles (Ne), the percentage of polymorphic loci (P%), mean gene diversity (He) and the gene flow (Nm) [52]. GenAlEx was also used to analyze molecular variance (AMOVA). AMOVA was used to partition the total genetic variation among individuals within the populations and between populations within a habitat. R version 4.1.2 was used to construct regression ANOVA and assess the association between the hordein bands and agronomic traits using the *lm* function. In the regression analysis, the agronomic traits were considered as an outcome variable and the hordein bands as a predictor variable. Stepwise regression analysis was also computed to further identify those bands that should remain in the multiple regression analysis model using the *olsrr* function to best explain the association.

## Supplementary Information

**Additional file 1:** **Table S1.** Estimates of the range, range unit and mean along with standard errors computed using pooled data of the six environments for the nine quantitative traits used.

**Additional file 2:** **Table S2-1.** Best linear unbiased predictor mean for grain yield (ton ha^-1^)of 19 six rowed barley lines pooled over years within the location and overall environments. **Table S2-2. **The mean performance of 19 barley lines for eight pheno-agronomic traits combined across six environments.

**Additional file 3:** **Table S3.** Pairwise Population Matrix of Nei’s Genetic Distance (below diagonal) and Nei’s Genetic Identity (above diagonal) based on 2 hordein loci between six-rowed barley lines.

**Additional file 4:** **Table S4.** Correlation among the independent variables (hordein bands) based on grain yield.

**Additional file 5:** **Table S5.** List of the barley lines included in the study, their geography of origin and caryopsis type.

## Data Availability

Estimates of the range, range unit and mean along with standard errors computed using pooled data of the six environments for the nine quantitative traits used summarized in Table S1. Best linear unbiased predictor mean for grain yield (ton/ha) of 19 six rowed barley lines pooled over years within the location and overall environments is provided in Table S2-1. The mean performance of 19 barley lines for eight pheno-agronomic traits combined across six environments provided in Table S2-2. Pairwise population matrix of Nei's genetic distance and genetic identity based on two hordein loci between six rowed barley lines summarized in Table S3. Correlation among the independent variables (hordein bands) based on grain yield provided in Table S4. Passport dat of 18 barley lines developed from farmers varieties their treatment number, accession number, geographic origin/zone, latitude, longitude, altitude of collection of the varieties and caryopsis type are summarized in Table S5.

## Abbreviations

DH: Days to heading
DM: Days to maturity
GFP: Grain filling period
PHT: Plant height
NET: Number of effective tillers
SPL: Spike length
NK: Number of kernel
TKW: Thousand kernel weight
GY: Grain yield
SDS-PAGE: Sodium dodecyl sulphate polyacrylamide gel electrophoresis
ANOVA: Analysis of variance
REML: Restricted maximum likely hood
masl: Meters above sea level
