# Supplementary material for: The association between hordein polypeptide banding and agronomic traits in partitioning genetic diversity in six-rowed Ethiopian barley lines (Hordeum vulgare L.)
Source: BMC Plant Biol. 2023 Feb 20;23:102. doi: 10.1186/s12870-023-04117-x (PMC9940401; doi:10.1186/s12870-023-04117-x)
Supplement: Supplementary file 1 — Additional file 1: Table S1. Estimates of the range, range unit and mean along with standard errors computed using pooled data of the six environments for the nine quantitative traits used. [file 12870_2023_4117_MOESM1_ESM.docx]

Table S1 Estimates of the range, range unit and mean along with standard errors computed using pooled data of the six environments for the nine quantitative traits used.

| Traits | Range  (min to max) | Range unit | Grand mean± SE |
| --- | --- | --- | --- |
| DH | 69.74-81.16 | 11.42 | 75.62±2.81 |
| DM | 107.65-117.44 | 9.79 | 112.57±2.61 |
| GFP | 35.62-39.34 | 3.72 | 36.94±0.44 |
| PHT | 92.26-104.40 | 12.14 | 98.05±2.52 |
| NET | 2.00-2.16 | 0.16 | 2.08±0.00 |
| SPL | 5.40-6.85 | 1.45 | 6.12±0.06 |
| NK | 42.91-51.08 | 8.17 | 46.76±2.06 |
| TKW | 32.54-41.99 | 9.45 | 38.87±2.08 |
| GY | 1.87-2.97 | 1.11 | 2.51±0.03 |

DH= Days to heading, DM= Days to maturity, GFP=Grain filling period, PHT=Plant height, NET= Number of effective tillers, SPL=Spike length, NK= Number of kernel, TKW= Thousand kernel weight, GY=Grain yield.
