# Supplementary material for: The association between hordein polypeptide banding and agronomic traits in partitioning genetic diversity in six-rowed Ethiopian barley lines (Hordeum vulgare L.)
Source: BMC Plant Biol. 2023 Feb 20;23:102. doi: 10.1186/s12870-023-04117-x (PMC9940401; doi:10.1186/s12870-023-04117-x)
Supplement: Supplementary file 2 — Additional file 2: Table S2-1. Best linear unbiased predictor mean for grain yield (ton ha-1)of 19 six rowed barley lines pooled over years within the location and overall environments. Table S2-2. The mean performance of 19 barley lines for eight pheno-agronomic traits combined across six environments. [file 12870_2023_4117_MOESM2_ESM.docx]

Table S2-1. Best linear unbiased predictor mean for grain yield (ton ha^-1^) of 19 six rowed barley lines pooled over years within the location and overall environments.

| Rank | Arsi Negelle | | Chefedonsa | | Holleta | | Pooled over environments | | |
| --- | --- | --- | --- | --- | --- | --- | --- | --- | --- |
|  | Genotype | Mean | Genotype | Mean | Genotype | Mean | Genotype Mean | | |
| 1 | 17146-9 | 3.15 | 16811-6 | 1.93 | 16811-6 | 3.60 | 16811-6 | 2.97 | |
| 2 | 17148-16 | 2.78 | 17206-11 | 1.88 | 16814-7 | 3.53 | 16814-7 | 2.92 | |
| 3 | HAR-1307 | 2.75 | 16910-19 | 1.83 | 16812-4 | 3.42 | 17146-9 | 2.84 | |
| 4 | 16734-6 | 2.69 | 16810-13 | 1.83 | 17204-5 | 3.28 | 16734-6 | 2.81 | |
| 5 | 16814-7 | 2.68 | 17204-5 | 1.82 | 16734-6 | 3.26 | 17204-5 | | 2.78 |
| 6 | 16820-16 | 2.62 | 17146-9 | 1.80 | 16820-16 | 3.24 | 16812-4 | | 2.76 |
| 7 | 16811-6 | 2.58 | 16734-6 | 1.80 | HAR-1307 | 3.18 | 16820-16 | | 2.76 |
| 8 | 17204-5 | 2.55 | 16863-2 | 1.79 | 17146-9 | 3.03 | HAR-1307 | | 2.75 |
| 9 | 16824-15 | 2.46 | 16812-4 | 1.75 | 16910-19 | 3.01 | 17148-16 | | 2.62 |
| 10 | 16810-13 | 2.44 | 16822-12 | 1.73 | 16810-13 | 2.91 | 16810-13 | | 2.57 |
| 11 | 16812-4 | 2.30 | 16814-7 | 1.71 | 16822-12 | 2.90 | 16910-19 | | 2.55 |
| 12 | 16910-19 | 2.22 | 16956-11 | 1.69 | 17148-16 | 2.88 | 16824-15 | | 2.49 |
| 13 | 16822-12 | 2.17 | 16820-16 | 1.67 | 16824-15 | 2.85 | 16822-12 | | 2.47 |
| 14 | 16956-11 | 2.03 | 16809-14 | 1.62 | 16956-11 | 2.61 | 16956-11 | | 2.27 |
| 15 | 16863-2 | 2.01 | HAR-1307 | 1.62 | 16809-14 | 2.56 | 16809-14 | | 2.14 |
| 16 | 17206-11 | 2.00 | 17240-1 | 1.62 | 17244-19 | 2.24 | 16863-2 | | 2.11 |
| 17 | 17240-1 | 2.00 | 17148-16 | 1.61 | 16863-2 | 2.23 | 17244-19 | | 2.03 |
| 18 | 17244-19 | 1.94 | 16824-15 | 1.51 | 17240-1 | 1.99 | 17240-1 | | 1.95 |
| 19 | 16809-14 | 1.72 | 17244-19 | 1.42 | 17206-11 | 1.70 | 17206-11 | | 1.87 |
| LSD |  | **0.17** |  | **0.14** |  | **0.33** |  | | **0.30** |
| CV |  | **9.06** |  | **8.05** |  | **17.96** |  | | **6.45** |

LSD= least significant difference, CV= coefficient of variation

Table S2-2 The mean performance of 19 barley lines for eight pheno-agronomic traits combined across six environments

| Genotype | Origin | DH | DM | GFP | PHT | TIL | SPL | NK | TKW |
| --- | --- | --- | --- | --- | --- | --- | --- | --- | --- |
| 16811-6 | Hadiya | 74.74 | 110.96 | 36.48 | 98.39 | 2.04 | 5.55 | 49.03 | 39.85 |
| 16814-7 | Hadiya | 76.12 | 112.33 | 36.48 | 96.75 | 2.02 | 5.66 | 49.05 | 40.16 |
| 17146-9 | Arsi | 73.90 | 111.50 | 37.34 | 103.98 | 2.01 | 6.36 | 51.08 | 41.49 |
| 16734-6 | Guraghe | 74.09 | 110.02 | 36.31 | 97.93 | 2.13 | 5.51 | 47.54 | 38.76 |
| 17204-5 | North Gonder | 81.16 | 117.44 | 36.54 | 97.51 | 2.16 | 6.85 | 48.68 | 41.02 |
| 16812-4 | Hadiya | 75.71 | 111.82 | 36.42 | 98.22 | 2.07 | 5.49 | 47.63 | 39.75 |
| 16820-16 | Guraghe | 75.57 | 111.68 | 36.42 | 97.76 | 2.04 | 5.40 | 49.90 | 38.19 |
| HAR-1307 | Cultivar | 74.31 | 115.09 | 39.34 | 98.14 | 2.13 | 6.20 | 42.92 | 41.27 |
| 17148-16 | Arsi | 77.32 | 117.07 | 38.70 | 98.43 | 2.13 | 6.25 | 44.66 | 41.99 |
| 16810-13 | Hadiya | 78.02 | 113.94 | 36.31 | 97.00 | 2.05 | 5.53 | 45.96 | 38.84 |
| 16910-19 | Arsi | 81.16 | 115.97 | 35.62 | 104.40 | 2.07 | 6.49 | 44.05 | 39.84 |
| 16824-15 | Guraghe | 78.94 | 115.69 | 36.83 | 101.67 | 2.03 | 6.58 | 47.06 | 41.78 |
| 16822-12 | Guraghe | 76.21 | 114.45 | 37.75 | 97.47 | 2.00 | 5.86 | 50.39 | 39.02 |
| 16956-11 | Arsi | 72.52 | 108.78 | 36.51 | 92.26 | 2.14 | 6.32 | 45.51 | 32.54 |
| 16809-14 | Hadiya | 72.42 | 107.72 | 35.91 | 95.41 | 2.10 | 6.35 | 44.13 | 36.40 |
| 16863-2 | Arsi | 79.05 | 115.49 | 36.62 | 99.43 | 2.07 | 6.29 | 46.84 | 41.52 |
| 17244-19 | Awi | 70.34 | 107.65 | 37.16 | 96.28 | 2.14 | 6.66 | 43.60 | 36.37 |
| 17240-1 | Awi | 69.74 | 109.47 | 38.67 | 97.21 | 2.13 | 6.51 | 47.46 | 34.28 |
| 17206-11 | North Gonder | 75.57 | 111.68 | 36.42 | 94.74 | 2.07 | 6.39 | 42.91 | 35.45 |

DH= Days to heading, DM= Days to maturity, GFP=Grain filling period, PHT=Plant height, TIL= Number of fertile tillers, SPL=Spike length, NK= Number of kernel, TKW= Thousand kernel weight, GY=Grain yield.
