# Supplementary material for: The association between hordein polypeptide banding and agronomic traits in partitioning genetic diversity in six-rowed Ethiopian barley lines (Hordeum vulgare L.)
Source: BMC Plant Biol. 2023 Feb 20;23:102. doi: 10.1186/s12870-023-04117-x (PMC9940401; doi:10.1186/s12870-023-04117-x)
Supplement: Supplementary file 3 — Additional file 3: Table S3. Pairwise Population Matrix of Nei’s Genetic Distance (below diagonal) and Nei’s Genetic Identity (above diagonal) based on 2 hordein loci between six-rowed barley lines. [file 12870_2023_4117_MOESM3_ESM.docx]

Table S3 Pairwise Population Matrix of Nei’s Genetic Distance (below diagonal) and Nei’s Genetic Identity (above diagonal) based on 2 hordein loci between six-rowed barley lines.

| Population ID | Awi | Arsi | Guraghe | Hadiya | North Gonder |
| --- | --- | --- | --- | --- | --- |
| Awi | ** | 0.755 | 0.468 | 0.605 | 0.513 |
| Arsi | 0.280 | ** | 0.833 | 0.902 | 0.913 |
| Guraghe | **0.759** | 0.182 | ** | **0.977** | 0.913 |
| Hadiya | 0.503 | 0.103 | 0.023 | ** | 0.906 |
| North Gonder | 0.668 | 0.091 | 0.091 | 0.099 | ** |
|  |  |  |  |  |  |
