# Supplementary material for: The association between hordein polypeptide banding and agronomic traits in partitioning genetic diversity in six-rowed Ethiopian barley lines (Hordeum vulgare L.)
Source: BMC Plant Biol. 2023 Feb 20;23:102. doi: 10.1186/s12870-023-04117-x (PMC9940401; doi:10.1186/s12870-023-04117-x)
Supplement: Supplementary file 4 — Additional file 4: Table S4. Correlation among the independent variables (hordein bands) based on grain yield. [file 12870_2023_4117_MOESM4_ESM.docx]

Table S4 Correlation among the independent variables (hordein bands) based on grain yield

|  | GY | M62 | M60 | M58 | M52 | M50 | M48 | M48a | M48b | M48c | M47 | M46a | M46B |
| --- | --- | --- | --- | --- | --- | --- | --- | --- | --- | --- | --- | --- | --- |
| GY | 1 |  |  |  |  |  |  |  |  |  |  |  |  |
| M62 | -0.334 | 1.000 |  |  |  |  |  |  |  |  |  |  |  |
| M60 | -0.302 | -0.102 | 1.000 |  |  |  |  |  |  |  |  |  |  |
| M58 | 0.624 | -0.248 | -0.456 | 1.000 |  |  |  |  |  |  |  |  |  |
| M52 | -0.629 | 0.456 | -0.224 | -0.544 | 1.000 |  |  |  |  |  |  |  |  |
| M50 | 0.307 | -0.081 | 0.322 | -0.018 | -0.177 | 1.000 |  |  |  |  |  |  |  |
| M48 | 0.217 | -0.276 | 0.077 | 0.685 | -0.606 | -0.402 | 1.000 |  |  |  |  |  |  |
| M48a | 0.433 | -0.347 | 0.294 | 0.716 | -0.760 | 0.233 | 0.797 | 1.000 |  |  |  |  |  |
| M48b | 0.433 | -0.347 | 0.294 | 0.716 | -0.760 | 0.233 | 0.797 | 1.000 | 1.000 |  |  |  |  |
| M48c | 0.543 | -0.394 | 0.259 | 0.630 | -0.864 | 0.205 | 0.701 | 0.880 | 0.880 | 1.000 |  |  |  |
| M47 | 0.078 | -0.056 | -0.102 | -0.248 | -0.122 | -0.081 | -0.276 | -0.347 | -0.347 | -0.394 | 1.000 |  |  |
| M46a | -0.629 | 0.456 | -0.224 | -0.544 | 1.000 | -0.177 | -0.606 | -0.760 | -0.760 | -0.864 | -0.122 | 1.000 |  |
| M46B | -0.629 | 0.456 | -0.224 | -0.544 | 1.000 | -0.177 | -0.606 | -0.760 | -0.760 | -0.864 | -0.122 | 1.000 | 1.000 |

GY= grain yield, M62= band 62, M60=band 60, M58=band 58, M50=band 50, M48=band 48, M48a=band 48a, M48b=band 48b, M48c=band 48c, M47=band47, M46a=band 46a, M46b=band46b
