# Supplementary material for: The association between hordein polypeptide banding and agronomic traits in partitioning genetic diversity in six-rowed Ethiopian barley lines (Hordeum vulgare L.)
Source: BMC Plant Biol. 2023 Feb 20;23:102. doi: 10.1186/s12870-023-04117-x (PMC9940401; doi:10.1186/s12870-023-04117-x)
Supplement: Supplementary file 5 — Additional file 5: Table S5. List of the barley lines included in the study, their geography of origin and caryopsis type. [file 12870_2023_4117_MOESM5_ESM.docx]

Table S5 List of the barley lines included in the study, their geography of origin and caryopsis type.

| Treatment no. | Accession number | Geography of origin/ Zone of collection | Latitude | Longitude | Altitude | Caryopsis type |
| --- | --- | --- | --- | --- | --- | --- |
| 1 | 16734-6 | Guraghe | 07-57-09-N | 38-04-09-E | 2905 | Hulled |
| 2 | 16809-14 | Hadiya | 07-40-07-N | 38-48-03-E | 2525 | Hulless |
| 3 | 16810-13 | Hadiya | 07-40-09-N | 37-48-03-E | 2525 | Hulled |
| 4 | 16811-6 | Hadiya | 07-42-50-N | 37-49-24-E | 2735 | Hulled |
| 5 | 16812-4 | Hadiya | 07-42-50-N | 37-49-22-E | 2762 | Hulled |
| 6 | 16814-7 | Hadiya | 07-42-50-N | 37-49-19-E | 2770 | Hulled |
| 7 | 16820-16 | Guraghe | 07-58-02-N | 37-57-00-E | 2722 | Hulled |
| 8 | 16822-12 | Guraghe | 07-55-44-N | 37-57-27-E | 2824 | Hulled |
| 9 | 16824-15 | Guraghe | 07-55-40-N | 37-56-28-E | 2818 | Hulled |
| 10 | 16863-2 | Arsi | 08-23-15-N | 38-26-20-E | 3070 | Hulled |
| 11 | 16910-19 | Arsi | 07-14-00-N | 38-57-34-E | 2821 | Hulled |
| 12 | 16956-11 | Arsi | 07-36-32-N | 39-16-34-E | 2873 | Hulless |
| 13 | 17146-9 | Arsi | 07-31-52-N | 38-57-11-E | 2660 | Hulled |
| 14 | 17148-16 | Arsi | 07-31-53-N | 38-57-09-E | 2656 | Hulled |
| 15 | 17204-5 | North Gonder | 13-10-32-N | 37-58-06-E | 2969 | Hulled |
| 16 | 17206-11 | North Gonder | 13-12-16-N | 37-58-24-E | 3202 | Hulled |
| 17 | 17240-3 | Agew Awi | 10-56-13-N | 36-53-51-E | 2514 | Hulless |
| 18 | 17244-19 | Agew Awi | 10-50-11-N | 36-50-55-E | 2521 | Hulless |
| 19 | HAR-1307 | Improved/cultivar | 07-57-09-N | 38-04-09-E | 2905 | Hulled |
